# First Study on profiling of gut microbiome in wild and captive Sumatran orangutans (*Pongo abelii*)

**DOI:** 10.14202/vetworld.2023.717-727

**Published:** 2023-04-12

**Authors:** Safika Safika, Agustin Indrawati, Usamah Afiff, Yohana Tri Hastuti, Zureni Zureni, Afif Pranaya Jati

**Affiliations:** 1 Division of Medical Microbiology, School of Veterinary Medicine and Biomedical Sciences, IPB University, Bogor, Indonesia; 2 Senior Veterinarian, Taman Safari Indonesia, Bogor, Indonesia; 3 Class II Agricultural Quarantine Center Medan, Indonesia; 4 Indonesian Society of Bioinformatics and Biodiversity Indonesia

**Keywords:** captive Sumatran orangutans, core microbiome, gut microbiota, *Pongo abelii*, wild Sumatran orangutans

## Abstract

**Background and Aim:**

Orangutans are an “umbrella species” for conserving tropical forests in Sumatra and Kalimantan. There are remarkable changes between the gut microbiomes of wild and captive Sumatran orangutans. This study aimed to profile gut microbiota of wild and captive Sumatran orangutans.

**Materials and Methods:**

Nine fecal samples collected from wild orangutans and nine fecal samples collected from captive orangutans were divided into three replicates. Each replicate randomly combined three pieces and were analyzed on the Illumina platform. A bioinformatics study of 16S rRNA according to Qiime2 (Version 2021.4) and microbiome profiling analysis was conducted.

**Results:**

The relative abundance of different microbial taxa varied significantly between wild and captive Sumatran orangutans. Among the operational taxonomic units, various proportions of *Firmicutes*, *Proteobacteria*, *Bacteroidetes*, *Euryarchaeota*, *Acidobacteria, Actinobacteria* and *Verrucomicrobia* predominated. *Solobacterium* was found only in 19% of captive orangutans. *Methanobrevibacter* was identified to be prevalent among wild orangutans (16%). Analysis of the core microbiome from the combined wild and captive data revealed seven species as cores. According to linear discriminant analysis effect size, *Micrococcus luteus*, *Bacteroidescaccae*, *Lachnospiraceae bacterium*, *Ruthenibacterium lactatiformans*, *Haemophilus haemolyticus*, and *Chishuiella* spp. were microbiome biomarkers in captive orangutans, whereas *Roseburia inulinivorans*, *Collinsella aerofaciens*, *Oscillibacter* spp., and *Eubacterium hallii* were microbiome biomarkers in wild orangutans.

**Conclusion:**

There were differences in the microbiome biomarkers of wild and captive Sumatran orangutans. This study is important for understanding the role of gut bacteria in the health of Sumatran orangutans.

## Introduction

In general, the gut microbiota of each primate is distinct [1, 2]. Diet, digestive physiology, and phylogenetics are frequently necessary to produce a particular bacterial population [3]. The gut microbiota is composed of microorganisms that reside in the digestive tract [4, 5]. Its composition varies biogeographically, resulting in communities that are environment-specific. Several factors, including nutrition [6], environment [7], stress [8], and drugs [9], influence the gut microbiota. Diet is considered the most influential factor in the evolution of primates, affecting their anatomy, physiology, and ecology [10]. Researchers believe that environmental and dietary factors influence the microbiota of primates [11, 12].

Orangutans are an “umbrella species” for conserving tropical forests in Sumatra and Kalimantan [13]. Orangutans are the only species of giant ape found in Asia. In comparison, three of its closest relatives reside in Africa, including gorillas, chimpanzees, and bonobos. In Indonesia, three orangutan species exist, including *Pongo pygmaeus* in Kalimantan, *Pongo abelii* in Sumatra, and *Pongo tapanuliensis* in Tapanuli [14]. The orangutan population is currently facing a significant decline. Under criterion A4bcd, the International Union for Conservation of Nature [15] Red List of Threatened Species has placed orangutans in the group of endangered animals with the status “Critically Endangered.” Each year, the population of Sumatran orangutans is diminishing. It is estimated that there are approximately 14,470 orangutans in Indonesia [16]. This reduction is due to the conversion of forest functions, illegal logging, and mining, which destroy the environment of orangutans. These environmental changes damage the gut ecosystem by generating an imbalance in the gut ecology. Several projects have been launched to protect the orangutan population, including *in situ* and *ex situ* conservation. In situ conservation is used to preserve orangutans in their native habitat. The objective of this strategy is to monitor orangutan conservation activities and their habitats [17]. It is possible to engage in *in situ* conservation, area stability, corridor development, and the transition of nonforest cultivation zones into protected areas to conserve orangutans in their habitat. *Ex situ* conservation refers to the maintenance and protection of orangutans in zoos and wildlife parks, such as the Indonesian Safari Park, which is home to one of Indonesia’s captive species [16]. The Sumatran orangutan consumes up to 400 different foods in its natural habitat, including young leaves, sap, flowers, honey, shoots, stems, seeds, nuts, bamboo, mushrooms, pith, bark, dirt, termites, ants, eggs, and invertebrates [18]. Orangutans are opportunistic eaters, consuming anything they can obtain, including honey from beehives. Sumatran orangutans are always on the move in the quest for their favorite food due to their affinity for unusual foods and randomly spread in their habitat [13]. Orangutans utilize distinctive mobility patterns to obtain the materials required for survival and reproduction [18]. Captive breeding can modify gut microbiota populations [4, 7]. For instance, primates in captivity may be fed commercial diets instead of natural foods, interact with humans, and obtain antibiotics, deworming drugs, and vaccines.

Several trends concerning the involvement of gut microbiota in health have been identified [19]. The microbiome bacteria aid digestion, control the immune system, defend against disease-causing bacteria, and generate vitamins such as the blood-clotting Vitamin K and the B Vitamins B12, thiamin, and riboflavin. Furthermore, intestinal bacteria are required to ferment non-digestible substrates, including food fibers and endogenous intestinal mucus. This fermentation encourages the growth of bacteria that generate short-chain fatty acids (SCFAs) and gases [20]. Moreover, a correlation exists between a decline in the diversity of gut bacteria and gastrointestinal (GI) disorders and inflammation. In addition, dysbiosis, or anomalies in the gut microbiota associated with ill health, can be produced by a reduction in normal flora, an increase in the abundance of pathogenic bacteria, or a change in the metabolic activity of the gut [21]. In their natural and captive environments, the gut microbiota of Sumatran orangutans can shed light on how captivity influences the gut microbiome of their health. This is crucial for highly endangered ape species such as Sumatran orangutans.

This study aimed to profile the gut microbiota of wild and captive Sumatran orangutans.

## Materials and Methods

### Ethical approval

This study obtained a permit and a written recommendation letter from Kementerian Lingkungan Hidup dan Kehutanan (KLHK) Direktorat Jenderal Konservasi Sumber Daya Alam dan Ekosistem (SK 433/KSDAE/SET.3/KSA.2/8/2021).

### Study period and location

This study was conducted in August 2022 at Gunung Leuser National Park, Southeast Aceh District and Taman Safari Indonesia, Bogor District.

### Sample collection

Fecal samples were collected at Gunung Leuser National Park in Southeast Aceh District from wild Sumatran orangutans and the Indonesian Safari Park from captive Sumatran orangutans. DNA extraction and polymerase chain reaction (PCR) were conducted at the bacteriology laboratory, School of Veterinary Medicine and Biomedical Sciences, IPB University, Bogor, Indonesia. Purification, library preparation, and sequencing were performed at Novogene Co., Ltd., Singapore.

The fecal samples were collected from wild and captive female adult Sumatran orangutans (*P. abelii*) aged 11–15 years that were in good health. Nine samples each from wild and captive orangutans were randomly combined into three replicates, each comprising three samples. Each orangutan’s feces were collected at the time of defecation in the morning after waking up, and those that fell to the ground were promptly picked from the middle to prevent contamination from the environment and placed in a plastic bag with a tight seal and plastic foil covering. The sample was maintained in a cold box at 2°C–10°C to prevent damage or contamination during transport. When the samples arrived at the bacteriology laboratory, they were placed in a refrigerator. Geographically, the size of Gunung Leuser National Park is 281,574.62 ha, spanning a latitude of 903°02′50.5″ north and a longitude of 097°25′02.0″ east. Regarding topography, the conditions range from coastal regions (0 m above mean sea level/masl) to mountainous areas (3000 masl). Approximately 80% of the region has a slope >40%. In this forest, free-roaming orangutans live naturally without human involvement in health management, nutrition, immunization, or worm treatment. Their diet consists of fruits collected and consumed directly from trees, and they are approximately 15–40 m off the ground, where feces are detected. Early in the morning, immediately after the orangutans defecated, the feces were collected [14].

Regarding the Indonesian Safari Park in Bogor, it is a 168-ha park located between 900 and 1800 masl and has an average temperature of 16°C–24°C. The orangutans living in captivity in this park were never administered antibiotics.

### DNA extraction and 16S rRNA analysis

The quality and quantity of data are affected by each stage in the process, from DNA sampling to final data collection, such as sample testing, PCR, purification, library preparation, and sequencing. However, data quality will directly affect the results of any subsequent information analysis. Therefore, quality control was performed at each procedural step to maintain the correctness and dependability of sequencing data.

### Bioinformatics of 16S rRNA

DNA libraries prepared using the NEBNext^®^ Ultra™ DNA Library Preparation Kit (Singapore) for Illumina and quantified using Qubit and Q-PCR were analyzed using the Illumina platform. The library was sequenced on an Illumina system, producing paired-end reads of 250 bp were assigned to samples and truncated by discarding the barcode and primer sequences. Combining paired-end readings with fast length adjustment of short reads FLASH (V1.2.11) (http://ccb.jhu.edu/software/FLASH/) [22]. According to Qiime2 (Version 2021.4) (https://view.qiime2.org) [23] quality-controlled procedure, the raw tags were subjected to quality filtering under precise filtering settings to produce high-quality clean tags. Using the UCHIME algorithm [24], the tags were compared with the reference database SILVA database (http://www.arb-silva.de/), and chimera sequences were eliminated [25]. The effective tags were subsequently obtained.

Using all effective tags, the sequences were analyzed using the Uparse software (Uparse v7.0.1090, http://drive5.com/uparse/) [26]. Similarity thresholds of 97% were used to assign sequences to the sameoperational taxonomic units (OTUs) identity on the Effective Tags of all samples, and then identified. The representative sequence of each OTU was evaluated for further annotation. Each representative sequence of Qiime2 (Version 2021.4) [27] was applied to the SSUrRNA database of the SILVA database (http://arb-silva.de/) [28] for species annotation at each taxonomic rank (Threshold: 0.81) [29] (kingdom, phylum, class, order, family, genus, and species) (http://arb-silva.de/). MUSCLE (Version 3.8.31; http://www.drive5.com/muscle/) can efficiently compare numerous sequences to determine the evolutionary relationship between all sample OTU sequences. Information on the abundance of OTUs was normalized using a standard sequence number corresponding to the sample, including minor sequences.

### Microbiome profiling analysis through a microbiome analyst

The OTU table generated by Qiime2 was submitted to MicrobiomeAnalyst https://www.microbiomeanalyst.ca/MicrobiomeAnalyst/home.xhtml. The OTU table and metadata adapted to the microbiomeAnalyst format for further analysis [30]. A low-number filter is created to avoid features with a minimal number in the sample due to sequencing errors or low-level contamination. Minimum count = 100, and based on the mean abundance value, the low variance filter is 20%, based on the interquartile range. Several criteria, including alpha diversity, beta diversity, and microbiome core, were profiled in this analysis of the community. Chao1, Shannon, and Simpson indices for alpha biodiversity were evaluated statistically by t-test/analysis of variance (ANOVA). Beta diversity represents the explicit comparison of microbial communities (in-between) based on their composition. Beta diversity is calculated for every pair of samples to generate a distance or dissimilarity matrix. Beta diversity can be performed using ordination based methods such as Principal Coordinates Analysis (PCoA) with, distance technique Bray-Curtis index in conjunction and statistical methods analysis of group analysis of similarities (ANOSIM) and permutational multivariate ANOVA (PERMANOVA).

The core microbiome had a sample prevalence of 20% and a relative abundance of 0.01%. Analysis of clustering and correlation using Heatmap Pearson is the clustering with distance measure; this parameter specifies how the distance between data points in the clustering input is measured. The linear discriminant analysis (LDA) using the linear discriminant analysis effect size (LEfSe) algorithm was applied for the comparison and classification of wild and captive orangutans.

## Results

### Sequencing results (histogram/table)

After quality screening with FastQC (Qiime2: https://view.qiime2.org), all read lacked primer and adapter sequences and were suitable for further analysis. The read count data indicated that Exsitu1 has a maximum of 156,192 reads and a minimum of 785,30 reads, both of which were from Sumatran orangutans in captivity (Table-1).

**Table-1:** Read counts from sample collections of wild and captive Sumatran orangutans (*Pongo abelii*).

Sample ID	Sample type	Read counts
Wild1	Wild orangutan	104,145
Wild2	Wild orangutan	999,34
Wild3	Wild orangutan	934,82
Exsitu1	Captive	156,192
Exsitu2	Captive	148,671
Exsitu3	Captive	785,30

### Microbial composition between wild and captive Sumatran orangutans

**Figure-1:**
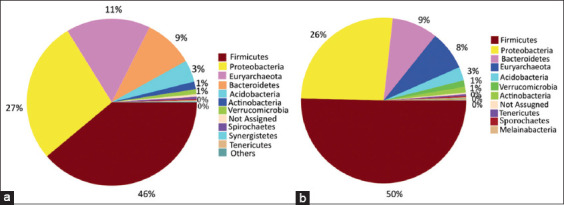
Taxa Abundance Profiling bacterial community structure of wild and captive Sumatran orangutans (*Pongo abelii*) based on 16S rRNA gene tag sequencing. (a) Taxa abundance in phylum wild orangutans; (b) Taxa abundance in phylum captive orangutans.

**Figure-2:**
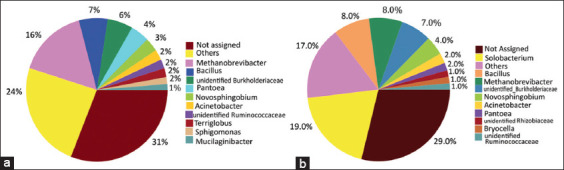
Taxa Abundance Profiling bacterial community structure of wild and captive Sumatran orangutans (*Pongo abelii*) based on 16S rRNA gene tag sequencing. (a) Taxa abundance in wild genus orangutans; (b) Taxa abundance in captive genus orangutans.

The relative abundance of various microbial taxa varied significantly between wild and captive Sumatran orangutans. *Firmicutes*, *Proteobacteria*, *Bacteroidetes*, *Euryarchaeota*, and *Acidobacteria* were the five phyla dominating Sumatran orangutans in the wild and captive samples based on OTUs, with variable percentages between the groups (Figure-1). *Firmicutes* comprised 46% of the average relative abundance of bacteria in wild orangutan samples, followed by *Proteobacteria* (27%), *Euryarchaeota* (11%), *Bacteroidetes* (9%), and *Acidobacteria* (3%). *Firmicutes* was detected in 50% of captive orangutan samples, followed by *Proteobacteria* (26%), *Bacteroidetes* (9%), *Euryarchaeota* (8%), and *Acidobacteria* (3%). *Methanobrevibacter* accounted for 16% of the average relative abundance of genus taxa in wild Sumatran orangutans, followed by *Bacillus* (7%), unidentified *Burkholderiaceae* (6%), and *Pantoea* (4%). In captive orangutans, *Solobacterium* accounted for 19%, *Bacillus* for 8%, *Methanobrevibacter* for 8%, and unidentified *Burkholderiaceae* for 7% of samples (Figure-2).

### Alpha and beta diversity of Sumatran orangutans

**Figure-3:**
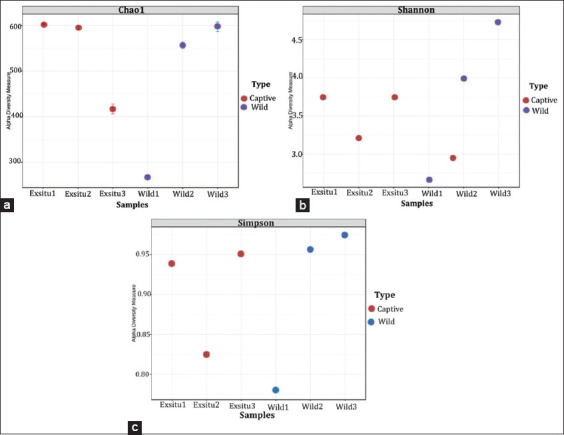
Alpha diversity (a) Chao1 index and (b) Shannon indeks (c) Simpson indeks. Comparing the microbial diversity. In wild and captive sumatran orangutans (*Pongo abelii*) (t-test/analysis of variance: *p = 0.62527).

The microbial communities in the guts of wild Sumatran orangutans were distinct from those of captive orangutans (Figure-3). Based on Chao1, Shannon, and Simpson diversity indices, no significant differences in the diversity and uniqueness of the bacteria were found between wild and captive Sumatran orangutans in terms of microbial diversity (Chao1’s p = 0.62527; t-test statistic = 0.53851; Shannon’s p = 0.73918; t-test statistic = −0.37456; and Simpson’s p = 0.99018; t-test statistic = 0.013241).

The Chao1 index extrapolates the number of unique taxa that may have been accounted for with more thorough sampling. The Chao1 index monitors and quantifies “richness” as the number of distinct species per sample. This score is a qualitative measure of alpha diversity and counts unique species. The Shannon diversity index considers the richness and evenness of species distribution. In contrast, the Simpson diversity index considers the number of species present and the relative abundance of each species [31].

Beta diversity is an exhaustive comparative evaluation of microbiomes based on their diversity. Therefore, beta diversity measurements are used to evaluate microbiome differences. A square matrix of “distance” or “dissimilarity” demonstrates the dissimilarity between samples. The Bray-Curtis dissimilarity analyses abundance data and calculates feature abundance differences.

**Figure-4:**
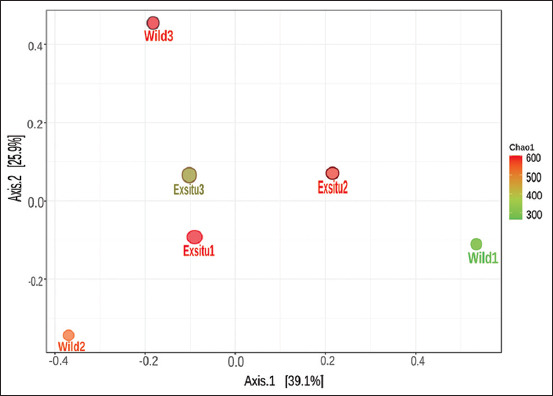
Beta diversity principle coordinate analysis wild and captive Sumatran orangutans (*Pongo abelii*) analysis of similarity R: −0.074074; p < 0.7.

Principal Coordinates Analysis can be used to illustrate beta diversity. Principle coordinate analysis optimizes the linear correlation between sample values. In this study, ANOSIM and PERMANOVA were used to determine statistical significance. Analysis of similarity demonstrated that rank was derived from the ordered distance between samples (R = 0.074074; p = 0.70). Analysis of similarity was conducted to establish whether the variation between groups is significantly greater than the variation within groups, which aids in evaluating the reason for group classification (Figure-4). However, the microbial community content of wild and captive Sumatran orangutans was not substantially different (p = 0.9) according to the PERMANOVA result.

### Core microbiome in Sumatran orangutans

The term “core microbiome” refers to the collection of taxa discovered in a significant proportion of the population above a certain abundance threshold. To perform this analysis, the count data are converted into compositional (relative) abundances.

**Figure-5:**
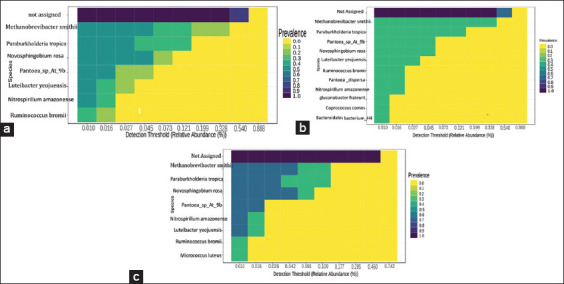
Core microbiome in the spesies of wild and captive sumatran orangutans (*Pongo abelii*). (a) combination of wild and captive; (b) wild sumatran orangutans; (c) captive sumatran orangutan.

Analysis of the core microbiome from the combined wild and captive data identified seven species as cores. Comparatively, if solely in the wild, 11 species became the core, whereas in captivity, nine species became the core. There is still much to be discovered regarding the data, as the highest-scoring data points were not assigned. *Methanobrevibacter smithii*, *Paraburkholderia tropica*, and *Novosphingobium rosa* often occur in wild and captive populations (Figure-5).

### Clustering and classification analysis of Sumatran orangutans

Heatmap clustering analysis with distance is a Pearson measure. The comparison and classification of wild and captive orangutans were performed using the effect size (LEfSe) method. A heatmap was generated using the abundance information of all samples to examine whether samples with comparable processing are clustered and to determine their identity and diversity.

**Figure-6:**
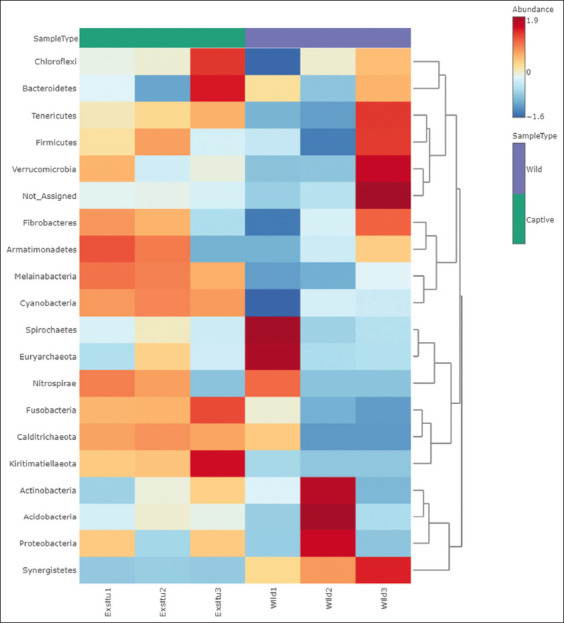
Taxonomic abundance cluster heatmap of wild and captive Sumatran orangutans (*Pongo abelii*). Wild1-wild3 are wild sumatran orangutans; Exsitu1-Exsitu3 captive sumatran orangutans. The mini heatmap to the right of the plot indicates whether the taxa are higher (red) or lower (blue) in each group.

In wild Sumatran orangutan, there was a predominance of *Verrucomicrobia*, *Firmicutes*, *Euryarchaeota*, *Spirochaetes*, *Proteobacteria*, *Acidobacteria*, and *Actinobacteria*. Intriguingly, unidentifiable bacteria detected in wild orangutan samples could not be assigned. *Bacteroidetes*, *Kiritimatiellaeota*, *Fusobacteria*, *Chloroflexi*, and *Armatimonadetes* were abundant in Exsitu3 and Exitu1 samples of captive orangutans (Figure-6).

**Figure-7:**
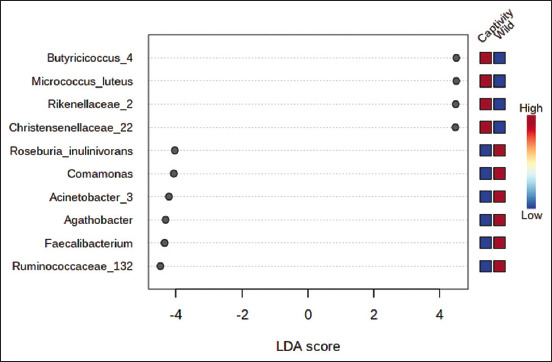
Ten of top features Graphical summary of LEfSe analysis of wild and captive Sumatran orangutans (*Pongo abelii*). The mini heatmap to the right of the plot indicates whether the taxa are higher (red) or lower (blue) in each group.

The LEfSe algorithm was designed for biomarker identification and metagenomics data interpretation. It applies the Kruskal-Wallis rank sum test to identify features with significant differential abundance concerning class labels, followed by LDA to evaluate the significance or magnitude of differentially abundant features [30]. *Micrococcus luteus*, *Bacteroides caccae*, *Lachnospiraceae bacterium*, *Ruthenibacterium lactatiformans*, *Haemophilus haemolyticus*, and *Chishuiella* spp. were the biomarkers of the microbiota of captive orangutans. In wild orangutans, the microbiome biomarkers were *Roseburia inulinivorans*, *Collinsella aerofaciens*, *Oscillibacter* spp., and *Eubacterium hallii* (Figure-7).

## Discussion

This research is a preliminary study and the first to compare the gut microbiota of wild and captive Sumatran orangutans, although there are significant limitations. The composition and diversity of the gut microbiome may differ among individuals with different characteristics, including sex, age, dietary preferences, geographic location, and health conditions. Male orangutans in the wild are more aggressive than females, making it more difficult to collect their feces. Sumatran orangutans are more arboreal than their counterparts in Kalimantan; they rarely descend to the ground. Instead, they are just above the tree branch. In the wild, orangutans create separate daytime and nighttime roosts for each tree, which are often close to their food source. During our sample collection from wild orangutans, we scoured the forest for trees that provided orangutans with food. After seeing the orangutans eating, we followed them till they defecated. The ability to distinguish between the gut microbiota of wild and captive orangutans by evaluating gut microbiota compositions, as demonstrated in the present study, is a significant advancement. This research is an initial step to understand the effect of the orangutan’s gut flora on its health. The microbiota in the orangutan’s gut sheds light on the dietary and digestive adaptations of this species. The differences between the microbiota of wild and captive Sumatran orangutans indicate that nutrition affects the health of orangutans.

We hypothesize that the gut microbiota of wild orangutans will be more diverse; a greater diversity of food sources in the forest will result in a significantly greater abundance of potentially pathogenic microbes in the normal gut flora. We did observe microbial diversity in the orangutans but did not investigate the incidence of potentially dangerous bacteria in captive orangutans. The gut microbiome exhibits remarkable changes between wild and captive Sumatran orangutans. Despite relatively uniform food and cleanliness, microbial diversity is reduced in captive orangutans. The caged orangutans in this experiment had never received antibiotic therapy. However, because the food is sourced from surrounding trees, captive orangutans can access a smaller variety of foods than their wild counterparts. Wild Sumatran orangutans can consume up to 400 different foods. In addition to flowers, young leaves, bark, honey, shoots, stems, seeds, nuts, bamboo, mushrooms, pith, bark, and various insects, 60% of their diet consisted of fruit. Orangutans frequently devour termites, ants, eggs, invertebrates, and anything else they can find, including honey from beehives, when the fruit is not in season. Orangutans also consume caterpillar larvae or cocoons as a source of protein at the beginning of the rainy season, when numerous caterpillars hatch [18].

Wild Sumatran orangutans exhibit more considerable OTU diversity and evenness and enormous species diversity. Several animal species such as *Rhinopithecus brelichi* [6], the Heliconius butterfly [32], turkeys [33], chimpanzees [34], and Grizzly bears [35] have exhibited alterations in their gut flora. According to OTU findings, there are differences between captive and wild orangutans. Compared with wild orangutans, whose fecal samples consisted of only 39% of *Firmicutes*, the samples of captive orangutans included 50% more of these species. However, the samples of wild orangutans (16%) contained more *Euryarchaeota* than those of captive orangutans (8%). Intriguingly, only 19.26% of the samples of captive orangutans contained the genus *Solobacterium*. *Methanobrevibacter* was discovered to prevail in 16.10% of orangutan samples in the wild. The genus *Solobacterium*, which was classified only in 2000, is derived from the Latin word for the sole and the word for tiny rod bacteria. These are Gram-positive, anaerobic, nonsporulating bacteria. *Solobacterium* was identified in human feces and were found to be phylogenetically separate from *Eubacterium*, *Holdemania*, and *Erysipelothrix* [36]. The genus was used to refer to a single bacterial species. The genus is now included in the family *Erysipelotrichaceae* of the phylum *Firmicutes*. *Solobacterium moorei* has been detected in periradicular lesions [37], endodontic infections [38, 39], and halitosis [40]. According to metagenomics research on the fecal microbiome and colorectal cancer, there exists a substantial link between several anaerobes [41], and *S. moorei* may be implicated in the development of colorectal cancer. *Methanobrevibacter* is an anaerobic archaea that is a member of the family *Methanobacteriaceae*. They produce methane, which serves as a methanogen for species that recycle carbon dioxide and hydrogen to produce methane. Archaeal methanogenesis increases the efficiency of fermented polysaccharides in animal intestines as bioreactors by preventing the production of hydrogen and other reaction byproducts. Most *Methanobrevibacter* species are found in the digestive tracts of mammals and humans [42]. *Methanobrevibacter* has been linked to a high-carbohydrate diet in humans. *Methanobrevibacter* has been detected in the digestive systems of big apes in the wild, cats, dogs, horses, rabbits, pigs, cattle, sheep, goats, and donkeys [43–47].

The core microbiome is extensively used in microbial ecology. The standard core microbiome identified the most abundant microbial species in the host population. The core microbiome refers to the set of microbial taxa or the genomic and functional features associated with those taxa, host traits, or environment of interest, with the host exceeding a given threshold of occupancy frequency [48]. Among the research that utilizes the core microbiome, there are studies of functional pathways, metabolic profiles, and functional genes [49, 50]. The presence of a typical core microbiome in the gut has enabled a greater understanding of the microbiome structure throughout the population of the host microbiome. The use of standard cores has increased our knowledge of the patterns of host–microbe interactions. The core microbiome bacteria include *M. smithii*, P. *tropica, N. rosa*, and *Pantoea* spp. According to the findings the present study, the core microbiome of wild orangutans contained 12 more species than those of captive orangutans (9 species). This may be due to the substantial differences in the gut microbiota between wild and captive Sumatran orangutans, resulting in more diversified gut microbiota in wild orangutans. Detecting the microbiome frequency patterns is a crucial starting point for understanding host–microbe interaction processes and provides candidates for future research into microbial function.

One of the species, *M. smithii*, can constitute 10% of all anaerobes in the colons of healthy people and is detected in the human intestine [42]. The cell wall of this archaea is composed of pseudopeptidoglycan, making it resistant to lysozyme. The genes in *M. smithii* are involved in the methanogenesis process, which uses CO_2_, H_2_, and formate. To accelerate the generation of adenosine triphosphate and SCFAs and hence increase the efficiency of the process, methanogenesis must utilize formate and hydrogen [51]. In addition, *M. smithii* produces the genes for proteins involved in the manufacture of cofactor vitamins that are required by enzymes in the methanogenic pathway, including the coenzyme Methionine synthase, riboflavin, and carriers of methyl groups (F430 and corrinoids). To use CO_2_, *M. smithii* also has an available route for the production of molybdopterin. The methanogenic enzymes Fwd (tungsten formylmethanofuran dehydrogenase), Hmd (methylene-H4MPT dehydrogenase), and Mcr (methyl-CoM reductase)] are constitutively expressed in the presence or absence of *Bacteroides thetaiotaomicron*. However, colonization significantly upregulates the expression of *RfaS*, an essential gene involved in methanopterin biosynthesis [52].

*Paraburkholderia* is a gram-negative bacterium that was first identified in 2004 as a nitrogen-fixing bacterium in sugarcane, maize, and teosinte plants. This bacterium originally belonged to the genus *Burkholderia*, which consisted of 96 species. However, 46 of these species have since formed the new genus *Paraburkholderia*. This genus contains nitrogen-fixing species and is isolated primarily from rhizosphere soils and plant tissues. *Paraburkholderia* *tropica* was initially discovered in the sugarcane stem [53, 54]. It is also known that these bacteria can produce biofilms in their surroundings [55]. Because Sumatran orangutans prefer to eat breadfruit (*Artocarpus altilis*), which has a high-carbohydrate content, it is possible that *P. tropica* is present in these primates.

*Novosphingobium* breaks down various xenobiotic substances and is linked to the biodegradation of environmental substrates such as lakes, soil, the ocean, wood, and sediments. According to the route metabolic profile, chromosomes house the majority (>62.5%) of genes involved in core carbon metabolism; nitrogen, phosphate, and sulfate metabolism; energy metabolism; and cell mobility. Between 21% and 50% of the genes involved in the degradation pathway are found in plasmids. The remarkable genomic and functional plasticity of *Novosphingobium* species allows them to reorganize their genomes in response to environmental changes and customize their metabolic profiles based on the substrates they encounter [56]. A member of the *Enterobacteriaceae* family, *Pantoea*, is found in the digestive tracts of humans and animals and in plants, soil, and water. These microorganisms are described as opportunistic pathogens and commensal bacteria. They are Gram-negative, coccoid to rod-shaped bacteria [57, 58]. According to reports, the genus *Pantoea* can cause postoperative meningitis in humans [59] and animal nosocomial infections in immunocompromised people [60].

A nonparametric statistical technique known as LEfSe can be used to identify the microbial taxa that differ significantly between groups. LDA was applied on taxa to determine the effect size that satisfied the significance level. Based on their LDA ratings, the taxa in this method are ranked in a tier list. The taxa that best represent each trait have an LDA score of 2; this method is frequently utilized [30]. According to the results of LEfSe, both captive and wild Sumatran orangutans have the same dominating functional bacteria in their guts. Although only a few samples are used currently, we attempted to investigate the gut microbiome as a biomarker in this study. *Micrococcus luteus, B. caccae, L. bacterium, R. lactatiformans, H. haemolyticus*, and *Chishuiella* spp. were discovered as microbiome biomarkers in captive orangutans in this study. In wild orangutans, *R. inulinivorans, C. aerofaciens, Oscillibacter* spp., and *E. hallii* were identified as microbiome biomarkers. Our study demonstrates the possibility of using gut microbiome data to identify several indicators that differ between wild and captive healthy Sumatran orangutans.

Locating recently defecated feces is challenging, and hence our study used only small samples of wild orangutans that inhabit tall trees and a population of approximately 30 wild orangutans that have been detected scattered over hundreds of hectares of forest. Furthermore, fecal samples may not accurately reflect the microbiota found in an animal’s digestive system. However, a previous study has reported no distinction between fecal microbiota and the bacteria in specific GI tracts [7]. The role of the gut microbiota community in metabolic activities and the relationship between microbiota and health and orangutan conservation management require further research.

## Conclusion

The abundance and diversity of the gut microbiota of wild Sumatran orangutans differ from those of captive orangutans. Only captive orangutans contained the genus *Solobacterium*. The core microbiome in the guts of Sumatran orangutans comprised *M. smithii*, *P. tropica*, *N. rosa*, and *Pantoea* spp. Microbiota biomarkers differed between wild Sumatran orangutans and those in captivity. Understanding the function of gut microbiota in the health of Sumatran orangutans is fundamentally based on the findings of this study.

## Authors’ Contributions

SS and AI: Conceptualization and writing-review and editing. SS: Methodology and writing-original draft preparation. SS, APJ, and UA: Software. SS, AI, UA, YTH, and ZZ: Validation. AU: Formal analysis. YTH: Investigation. ZZ: Resources and project administration. SS and UA: Data curation. UA: Visualization and APJ: Supervision. All authors have read, reviewed, and approved the final manuscript.

## Data Availability

The data supporting this study’s findings are available from the corresponding author on reasonable request.
